# Minimal clinically important difference of the 6-Minute walk test and daily step count at 3 months following surgery for lumbar spinal stenosis

**DOI:** 10.1007/s00586-025-09085-4

**Published:** 2025-07-15

**Authors:** Suzanne McIlroy, Yee Mah, Vassilios Tahtis, Abigail Beddard, Lindsay Bearne, John Weinman, Sam Norton

**Affiliations:** 1 King’s College Hospital NHS Foundation Trust, London, United Kingdom; 2 King’s College London, London, United Kingdom; 3 Imperial College London, London, United Kingdom; 4 City St George’s, University of London, London, United Kingdom

**Keywords:** MCID, Lumbar spinal stenosis, 6 minute walk test, Mean daily step count, Walking, Outcomes

## Abstract

**Purpose:**

This study aimed to estimate the minimal clinically important difference (MCID) in two objective measures of walking: 6-minute walk distance (6MWD) and mean daily step count in patients with lumbar spinal stenosis, three months post-surgery. Both anchor-based and distribution-based approaches were used to support result robustness and comparability.

**Methods:**

97 patients (mean age 70 ± 8.3 years; 50 female) were recruited from three UK hospitals. 6MWD (metres) and mean daily step count (measured over 7 days with an accelerometer), and self-rated clinical questionnaires were assessed pre- and 12-weeks post-surgery. The anchor-based method used the Oswestry Disability Index (ODI) and the satisfaction subscale of the Zurich Claudication Questionnaire. Receiver-operating characteristic (ROC) curve analysis was used to determine the optimal cutoff points for MCIDs for changes in the 6MWD and step count. The distribution-based method used 0.3 SD of the change scores.

**Results:**

Anchor-based MCIDs for the 6MWD were 26 m (ODI) and 35 m (walking satisfaction). The step count MCID could not be determined using the ODI but was 680 steps when anchored to satisfaction. The distribution-based method estimated MCIDs of 34 m for the 6MWD and 750 steps for step count.

**Conclusion:**

Even modest improvements in walking capacity and daily step count may be meaningful to patients recovering from LSS surgery. Further research is needed to validate the MCID for daily step count however, the identified MCIDs for the 6MWD (26–35 m) and daily step count (680–750 steps) provide practical thresholds for assessing meaningful change and can be used to inform goal setting within rehabilitation.

**Supplementary Information:**

The online version contains supplementary material available at 10.1007/s00586-025-09085-4.

## Introduction

Lumbar spinal stenosis (LSS) affects over 10% of the general population [1]. This degenerative condition characterised by narrowing of the spinal canal and/or neural foramina, compromises the associated neurovascular structures. A common symptom of LSS is neurogenic claudication- pain, weakness, and sensory disturbances in the lower limbs, sometimes accompanied by low back pain. Symptoms typically worsen with lumbar extension, and impact walking [2].

Reduced walking, quality of life, and independence prompt individuals to seek treatment including surgery [3]. Although surgery decompresses the spinal nerves, up to 40% of people report persistent walking disability [4].

Walking ability can be assessed through capacity and performance. Capacity refers to completing a specific task in a controlled setting, such as a timed walking test, whereas performance reflects real-world ability [5]. The 6-minute walk test measures capacity as the maximum distance (metres) walked in six minutes (6MWD). It is reliable and responsive to change in adults with long-term conditions, including LSS [6]. Step count, typically measured as mean steps-per-day over 5–7 days, is a measure of real-world walking performance [7]. Prior to assessing treatment effects in people with LSS, the clinical relevance of any changes must be established. The minimal clinically important difference (MCID) defines the smallest change patients consider meaningful [8].

MCIDs for 6MWD in chronic conditions typically range from 14 to 50 m (mean 30.5 m), depending on the population [9]. However, in people undergoing surgery for LSS, the MCID appears higher. To date, two studies in Japanese cohorts have examined this and estimated an MCID of 80–100 m at 12-months post-surgery [6, 10]. These exceed typical values in other chronic conditions, suggesting greater improvements may be needed to reflect meaningful recovery in LSS. Further research is required to determine whether these estimates are generalisable beyond Japanese populations and applicable across diverse demographics, cultural contexts, and healthcare systems [11].

Similarly, meaningful change in daily step varies across conditions, with estimates ranging between 560 and 1,000 steps/day in people with long term conditions [12, 13]. However, evidence for the MCID in LSS is limited. One study, also conducted in Japan, reported an MCID of 1,149 steps/day in conservatively treated LSS patients, which may not apply to those undergoing surgery [14]. Currently no study has established the MCID for daily step count in individuals undergoing LSS surgery.

Within the UK National Health System (NHS), spinal surgery patients are typically reviewed approximately 12 weeks post-operatively and discharged if there are no clinical concerns [15]. Calculating the MCID during this early post-operative period has clinical utility for evaluating improvement and identifying the need for additional support or rehabilitation.

This study aims to estimate MCID in the 6MWD and daily step count in patients with LSS 12 weeks after surgery. It uses an anchor-based approach, and a distribution-based approach to provide a comparative perspective and to validate the robustness of the results [8].

## Materials and methods

### Study design and population

This analysis utilised data from a prospective longitudinal study [16]. Ethics approval was obtained from the East Midlands-Nottingham 1 Research Ethics Committee (20/EM/0307). All participants provided written informed consent prior to enrolment.

### Participants

Between April 2021 and July 2022 consecutive patients from three NHS hospitals in England were identified. Inclusion criteria were: on a waiting list for elective decompressive surgery for degenerative LSS with neurogenic claudication, aged ≥ 50 years, conversational English or willing to use an interpreter. Exclusion criteria included: insufficient time for baseline data collection (e.g., surgery within one week of screening), LSS caused by tumour, fracture, or significant deformity (> 15^0^ lumbar scoliosis; grade II + spondylolisthesis); emergency surgery; >2 level instrumentation surgery; or other conditions causing walking restriction.

### Procedures

Potential participants were identified during surgical appointments or from clinic lists and invited to learn more either during the clinic or by phone. Those interested were mailed a participant information sheet, consent form, and a baseline demographic and clinical questionnaires. After returning consent forms, participants attended two assessment sessions: pre-surgery and 12 weeks post-surgery. Only those with data at both time points were included in the analysis; incomplete datasets were excluded without imputation.

### Measures

#### Walking capacity: 6-minute walk distance

Using a standardised protocol [17], participants were asked to walk as far as possible around two cones placed 10 m apart in a straight corridor for six minutes and the total distance walked (in metres) was recorded. This approach has previously been validated and found to be reliable and responsive to change in patients with LSS [18].

#### Walking performance: mean daily step count

Daily step count was measured with a triaxial accelerometer (ActivPal3™, PAL Technologies Ltd., Glasgow, UK). This device uses information about acceleration and thigh position to determine body posture, stepping, and cadence. Each participant wore the accelerometer on the mid-anterior thigh secured with a waterproof dressing. Data recording began at midnight of the assessment days, and participants were instructed to wear the device continuously for the next seven days, including when sleeping and bathing. Three-dimensional acceleration data was collected over 60 s epochs. Data were considered valid if the device was worn for ≥ 14 h per day for ≥ 5 days [19]. The use of an accelerometer as an objective outcome measure for physical activity in people with LSS is increasing, but the validity, reliability and responsiveness within this population has yet to be established.

### Anchors

Two anchor measures were used: the Oswestry Disability Index (ODI) as it is associated with walking capacity [20]; and post-operative satisfaction with walking ability as it directly reflects participant perceived improvement. The self-reported ODI is a validated, reliable, and responsive 10-item measure of back-related functional disability [21, 22]. Each item is rated on a 6-point Likert scales (0 = least, 5 = greatest disability) with scores expressed as a percentage—higher scores indicate greater disability. The MCID for the ODI has been reported to be 12.8 for LSS [5]. Satisfaction with post-operative walking ability was assessed using the walking ability item from the valid and reliable Zurich Claudication Scale [23]. Participants rated their satisfaction with their walking ability after surgery on a four-point Likert scale, ranging from ‘very satisfied’ to ‘very dissatisfied’. For analysis, we dichotomised responses into two categories: satisfied (combining ‘very satisfied’ and ‘somewhat satisfied’) and dissatisfied (combining ‘somewhat dissatisfied’ and ‘very dissatisfied’). As there was no neutral option on the scale we made the assumption that ‘somewhat satisfied’ reflected a meaningful improvement in walking ability. The other items from the satisfaction scale were not included, as they did not pertain specifically to walking ability.

### Statistical analysis

The two main approaches for calculating the MCID are anchor-based and distribution-based methods [24]. Anchor-based approaches compare changes in the outcome with an external criterion or measure of patient-perceived improvement, ensuring the calculation reflects clinically meaningful changes rather than statistical variation alone. Distribution-based approaches use statistical properties and distribution of outcome changes, assuming that changes in scores and statistical thresholds (e.g. standard deviations) represent meaningful change [24]. As no gold standard exists for calculating the MCID, multiple approaches are often used. We used the anchor based method as the primary approach and the distribution approach provides supporting information [8].

For the anchor-based approach, to define responders using the ODI, the change in ODI scores from baseline to the 12 week follow-up was calculated and dichotomised into responders and non-responders, depending on whether the change met or exceeded the established MCID threshold of 12.8. A receiver operating characteristic (ROC) curve was generated by varying the threshold change in 6MWD distance and step count, plotting sensitivity and specificity to detect responders based on the ODI-defined MCID. The optimal threshold was determined using Youden’s index, which balances sensitivity and specificity: J = sensitivity + specificity – 1. This approach was repeated using the satisfaction with walking scale, with responses dichotomised into *responders* (response ‘very satisfied’ and ‘somewhat satisfied’) and *non-responders* (response ‘very dissatisfied’ and ‘somewhat dissatisfied’).

For the distribution-based approach, the MCIDs were estimated using the standard deviations (SD) of the mean change values for the 6MWD and step count, with 0.3 SD and 0.5 SD being commonly used methods reported in the literature [8].

Baseline values between responders and non-responders were compared using the Mann-Whitney U test, as the data did not meet assumptions for parametric testing. Spearman’s rank correlation was used to assess the relationship between changes in the objective walking measures and the change in ODI and satisfaction with post-operative walking. A correlation coefficient of ≥ 0.30 is generally considered indicative of a satisfactory anchor [25].

Analyses were performed in Python using SciPy (https://scipy.org/) for statistical tests and Scikit-learn (https://scikit-learn.org/stable/) for AUC calculations.

### Sample size

An a-priori sample size calculation was not conducted for this study which was a secondary analysis of the longitudinal study cohort [16]. Therefore, we assessed the detectable effect size given the sample size available to ensure this allowed for valid analysis [26]. A sample of 97 participants equally divided into responders and non-responders provides 90% power to detect a difference with an effect size of 𝑑=0.58 with a two-sided 5% alpha. This corresponds to a mean difference of 70 m using a standard deviation of 120 m - approximately the estimated MCID of the 6MWD.

## Results

A total of 134 participants enrolled in the study. After excluding 17 who did not have surgery, 8 who were lost to follow-up, and 12 with no objective data (primarily due to concerns about COVID-19 infection or transport issues), 97 participants (47 male, 50 female, mean age 70 years, SD 8.3) were included. Participant characteristics are shown in Table 1. Two participants did not complete their post-operative ODI scores, and some accelerometers were not returned, leading to variation in sample size across different analyses. Tables 2 and 3 report the sample sizes for each outcome. Most participants had one level decompressed (*n* = 69, range 1–3). All participants had a laminectomy or laminotomy except one who had a transforaminal lumbar interbody fusion and decompression. Seven participants also had discectomies and five had foraminotomies. Median length of stay post-operatively was 1 day (range 0–16 days).

**Table 1:** Clinical and demographic characteristics of study participants (*n* = 97)

Demographic variable		*n* (%) or mean ± SD
Age (years)		70.0 ± 8.3
Sex	Male	47 (49%)
	Female	50 (52%)
Body mass index		29.0 ± 4.4
Education	Up to and including secondary school	63 (65%)
	Higher Education	34 (35%)
Employment status	Retired	69 (71%)
	Working	4 (4%)
	Unable to work	24 (25%)
Ethnicity	White British	74 (76%)
	Other White	8 (8.%)
	Black African	5 (5%)
	Black Caribbean	6 (6%)
	Asian	2 (2%)
	Other	2 (2%)
Current smoker		11 (11%)
Heart Disease		13 (13%)
Hypertension		54 (56%)
Diabetes		12 (12%)
Depression		9 (9%)
Rheumatoid arthritis		6 (6%)
Duration of LSS symptoms (months)		46 ± 42
Oswestry Disability Index	Pre-surgery	42.3 ± 15.0
6-minute walk test (m)	Pre-surgery	238.46 ± 112.1
	12-weeks post-surgery	299.84 ± 130.6
Mean daily step count	Pre-surgery	4879 ± 2488
	12-weeks post-surgery	5461 ± 3025

LSS: lumbar spinal stenosis; SD: standard deviation

**Table 2:** MCID thresholds for 6MWD, and differences in responders and non-responders based on the anchor approach

	Anchor: ODI (*n* = 95*)		Anchor: Satisfaction with walking (*n* = 97)	
	Baseline score	Change at 12-weeks post-operative	Baseline score	Change at 12-weeks post-operative
Responder: Non-responder, n	52:43		69:26	
Responder mean score (± SD)	229.20 m (99.40)	87.87 m (71.95)	239.38 m (110.76)	75.88 m (74.72)
Responder median score (IQR)	229.19 m (156.06)	84.51 m (100.93)	241.43 m (143.47)	69.20 m (111.00)
Non-responder mean score (± SD)	233.51 m (118.40)	31.67 m (60.43)	218.71 m (98.81)	26.73 m (51.76)
Non-responder median score (IQR)	235.60 m (175.10)	17.48 m (77.64)	203.44 m (143.47)	24.00 m (95.57)
MCID	26 m		35 m	
AUC	0.73		0.69	

*Missing ODI data in 2 participants

**Table 3:** MCID thresholds for step count, and differences in responders and non-responders based on the anchor approach

Step Count (*n* = 87*)	Anchor: ODI		Anchor: Satisfaction with walking	
	Baseline score	Change at 12-weeks post-operative	Baseline score	Change at 12-weeks post-operative
Responder: Non-responder, n	47:40		76:11	
Responder baseline mean score (± SD)	4978 (2401)	1026 (1947)	5170 (2503)	782 (1894)
Responder baseline median score (IQR)	4715 (3253)	615 (1447)	5261 (3818)	455 (1427)
Non-responder baseline mean score (± SD)	4754 (2527)	148 (1182)	4145 (2184)	226 (951)
Non-responder baseline median score (IQR)	4381 (3657)	20 (1237)	3996 (1885)	-9 (1103)
MCID	-245		678	
AUC	0.64		0.59	

*complete data for 87 participants

At baseline, there were no significant differences between the responder and non-responder groups either walking measure when anchored to the ODI (responders: 53 [55%], non-responders: 43 [45%]; 6MWD: *p* =.887, step count: *p* =.207) or walking satisfaction (responders: 69 [73%], non-responders: 26 [27%]; step count: *p* =.600; 6MWD: *p* =.372). Tables 2 and 3 summarise the mean and median baseline values and changes for each walking measure in both groups.

Changes in 6MWD and step count differed between responders and non-responders, with 6MWD showing greater separation between groups, despite some overlap. The distribution of changes was positively skewed among non-responders, and some participants exhibited changes comparable to responders. Across the cohort, the correlation between the change in 6MWD and change in ODI was 0.52 (*p* <.001), and satisfaction with post-operative walking was − 0.27 (*p* =.009). For step count, the correlations were 0.32 (*p* <.002) with ODI change and − 0.21 (*p* =.05) with satisfaction. For detailed visualisations, please see Supplementary Figs. 1–3.

### 6-minute walk distance MCID

**Fig. 1:**
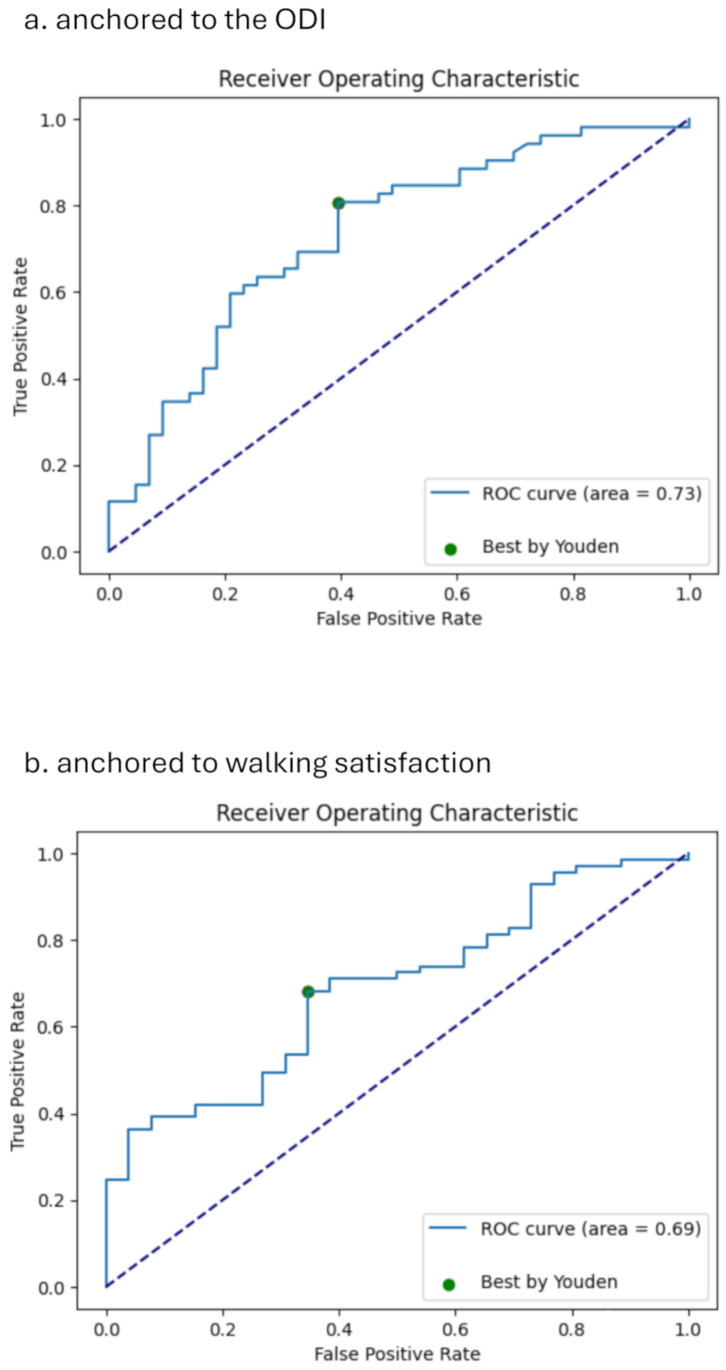
Receiver Operating Characteristic for 6 min walk distance (**a**) anchored to ODI; (**b**) anchored to walking satisfaction

The MCID estimates for the 6MWD, anchored to the ODI and walking satisfaction, are presented in Table 1. In the ROC curve analysis, the optimal threshold for the MCID that maximised sensitivity and specificity when anchored to the ODI was 26 m (specificity: 0.605, sensitivity: 0.808; Fig. 1a). When anchored to walking satisfaction, the optimal threshold was 35 m (specificity: 0.654, sensitivity: 0.681; Fig. 1b). The distribution-based approach estimated the 6MWD MCID as 34 m at 0.3SD and 56 m at 0.5SD.

### Mean daily step count MCID

**Fig. 2:**
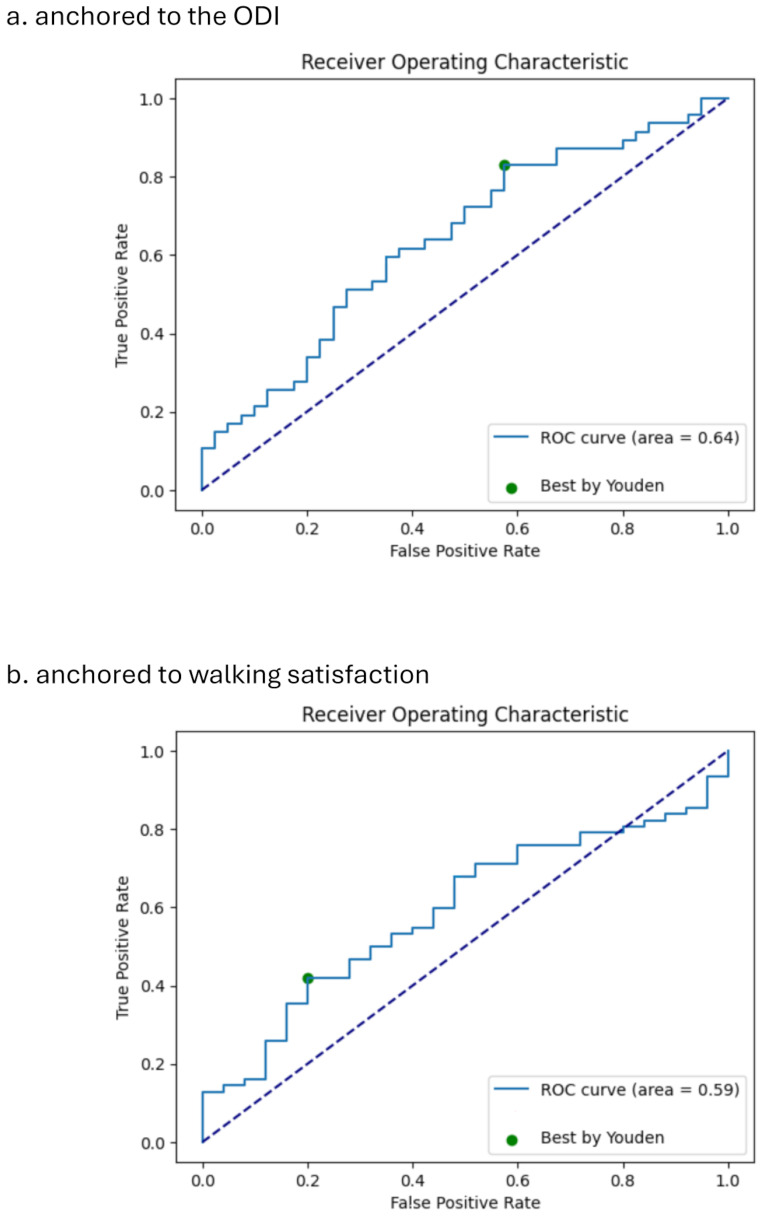
Receiver Operating Characteristic for mean daily step count (**a**) anchored to ODI; (**b**) anchored to walking satisfaction

The MCID estimates for step count, anchored to the ODI and walking satisfaction, are presented in Table 2. In the ROC curve analysis, the optimal threshold for the MCID that maximized sensitivity and specificity when anchored to the ODI was − 245 (specificity: 0.425, sensitivity: 0.830, Fig. 2a). When anchored to walking satisfaction, the optimal threshold was 678 (specificity: 0.80, sensitivity: 0.419; Fig. 2b). The distribution-based approach estimated the step count MCID as 746 at 0.3SD and 1244 at 0.5SD.

## Discussion

This study aimed to establish the MCID for both the 6MWD and mean daily step count in the early post-operative period in people undergoing surgery for LSS. For the 6MWD we identified MCIDs of 26 m and 35 m using ODI and walking satisfaction as anchors, respectively, aligning with the distribution-based estimate of 34 m and with previous research in other conditions [9]. We were unable to calculate the MCID for step count when using the ODI as an anchor, possibly because the ODI measures walking capacity rather than performance. However, using walking satisfaction as an anchor, the MCID was estimated as 678 steps, which aligned with the distribution-based approach of 746. Our findings offer valuable insights into meaningful walking improvements in patients’ post-surgery for LSS and contribute to the body of evidence surrounding the use of these measures in clinical practice. Importantly, our findings suggest that a relatively modest gain in walking capacity and performance can be perceived as meaningful by patients. The variability within the non-responder group, including some individuals who improved by more than 100 m in the 6MWD without reporting perceived benefit, highlights the subjective nature of patient-reported outcomes, the known discrepancy between subjective and objective outcome measures, and the need for multi-dimensional assessment tools.

The identified 6MWD MCID of 26–35 m is notably lower than reported by two studies with Japanese samples estimating an MCID of 80–100 m at 12-months post-surgery [6, 10]. However, our results are greater than the MCID for people with intermittent claudication caused by peripheral artery disease which produces similar leg pain [27]. Furthermore, our estimates align more closely with the systematic review which suggested a meaningful change of approximately 30 m across various populations [9]. A further study involving 692 individuals, including those with mobility disabilities, stroke survivors, and community-dwelling older adults, estimated a small but meaningful change in 6MWD of 20 m, while a substantial change was around 50 m [28]. The differences between the estimates in the Japanese samples and our findings potentially underscore the importance of considering cross-cultural variability when interpreting MCID values. Replication is important to validate the generalisability of these findings beyond the original cohort. It supports applicability across differing demographics, cultures, and healthcare systems [11].

The observed differences may also reflect differences in methodology, participant characteristics, or follow-up timing. A 10 m walkway was used in this study due to the unavailability of a longer, straight, flat corridor—an issue commonly encountered in clinical settings [29]. Using a 10 m walkway for the 6MWD can result in shorter walking distances compared to a 30 m walkway or continuous loop, primarily due to the increased number of turns disrupting walking rhythm [30]. However, some evidence suggests that the influence of walkway length on test outcomes diminishes with age, possibly due to the slower walking speeds inherent in older adults [29]. Therefore, the use of a 10 m walkway is clinically relevant and reflects real-world constraints often faced in routine practice.

In addition to cultural differences, participant characteristics varied between studies. The current cohort had a higher prevalence of heart disease (13% vs. 7%) than that reported by Takenaka and colleagues [6], and a longer mean symptom duration than both studies (4 vs. 1–2 years [6, 10]), potentially affecting physical capability. Surgical procedures and peri-operative care also differed: our cohort included almost no fusion procedures, whereas 41% of both other study participants underwent fusion. Length of stay was typically 1 day in our study, compared to 7 days, or 14 with fusion, in the study by Takenaka et al. Their patients received 40 min of daily inpatient physiotherapy, which may have supported earlier functional recovery, improved physical capacity, and reduced fear of movement, leading to greater 6MWD improvements. Future mediation analysis could help determine whether differences in peri-operative care, mediate the observed improvements in 6MWD.

Our assessments at 12 weeks post-surgery captured early improvements. At this stage, outcomes following surgery for LSS are predictive of long-term recovery [31], and most functional gains occur within the first 8–12 weeks and tend to plateau thereafter [32]. However, future research should determine consistency of MCID over longer periods.

The large variation and negative values in our MCID estimates for step count using ODI require careful consideration. MCID can be calculated using various methods, resulting in heterogeneity. As there is no consensus on the most appropriate approach [24], we followed common practice and applied multiple methods [8, 14]. The MCID values anchored to walking satisfaction and calculated using the distribution method were plausible, but the negative value anchored to the ODI was not. Given the consistency between the walking satisfaction anchored and distribution-based estimates we suggest an MCID of around 680 to 750 steps.

Three main factors likely explain the discrepancy for the ODI anchored estimate. First, although the ODI is reliable, valid, and responsive in LSS, it may lack sensitivity needed to detect changes specifically related to walking limitations [20]. As a composite measure of back-related disability, it may show improvement even if physical activity levels remain unchanged. Second, the ODI measures walking capacity rather than performance [20]. While we used the ODI due to its widespread use in spinal surgery it assesses a different construct and may not be a valid anchor for walking performance. A more specific walking performance measure should be used in future studies. Third, the negative value likely reflects that approximately 40% of the cohort walked fewer steps post-operatively than pre-operatively. People undergoing lumbar surgery often report fear of exercise whilst waiting for surgery, and lack of knowledge about how to manage their recovery and safely increase activity levels [33]. As a result, they may become deconditioned during the pre-operative period, which can continue or worsen in the peri-operative phase, and the observed reduction in step count may reflect this deconditioning. Some people may require a greater duration to increase their daily step count. However, findings of unchanged walking performance from 6 to 52 weeks post-surgery have been reported in other studies [7, 34, 35]. Notably, a study of 248 adults undergoing laminectomy for degenerative conditions found that those who walked at least 3500 steps per day at 6 weeks post-operative had 3.75 times higher odds of achieving significant pain and disability reduction at 1 year [36], underscoring the importance of early post-operative activity.

These results highlight the need for early access to rehabilitation and behaviour change programmes to improve walking capacity and performance and mitigate the negative consequences of low physical activity following surgery for LSS. The MCIDs calculated within the study can be used to inform goal setting in rehabilitation programmes in addition to serving as evaluation criteria for treatment efficacy. Furthermore, our results highlight that while a patient may report reduced pain this does not mean they are more physically active.

Our estimated step count MCID of 680–750 steps/day is lower than the estimate of 1,149 steps/day reported by Minetama and colleagues in conservatively managed LSS participants [14]. This discrepancy may be due to differences in calculation methods. Additionally, clinical differences between the study populations, such as surgical versus non-surgical status and variations in baseline scores, provide a plausible explanation for the differing results.

Intermittent claudication, caused by peripheral artery disease, has an MCID step count of 560–780 steps/day, which aligns with our findings. This is lower than Tudor-Locke et al.’s benchmark of 1,000 additional steps/day as a meaningful change in physical activity for older adults with disability and/or chronic illness [12]. While these comparisons provide context, the AUC, sensitivity, and specificity are poor to moderate, highlighting the need for further research to validate step count MCID thresholds specifically for post-LSS surgery populations. Given the low AUC values and weak correlation coefficients with both anchors, the MCID thresholds for daily step count should be interpreted with caution, as they may lack precision and reliability. Specifically, AUC values fell below the commonly accepted threshold of 0.70, and the anchors demonstrated only weak associations, limiting their robustness. The Physical Function scale of the Zurich Claudication Questionnaire [23], with its focus on walking capacity and performance, may offer a more relevant alternative anchor, however, a systematic review has reported inadequate criterion validity for this measure [22]. In the absence of a gold standard for defining MCID or classifying responders in step count, our estimates should be viewed as pragmatic starting points. Further research is needed to validate these thresholds and to identify more reliable anchors for future use.

### Strengths, limitations and future directions

To our knowledge this is the first study to investigate step count MCID in surgically treated patients with LSS and the first to assess 6MWD in the early post-operative period. Step count data were collected using research grade-accelerometers. Accelerometer use to objectively measure physical activity in people with LSS is increasing, but few studies have examined its validity, reliability, and responsiveness in this population. The relatively small sample may have limited the ability to detect subtle differences between responders and non-responders, particularly for step count. Additionally, large standard deviations in both the 6MWD and step count data underscore the heterogeneity of post-surgical outcomes in LSS patients. The low AUC values and low correlation coefficients between the walking measures and the anchors were below commonly accepted norms and therefore limit the confidence in the accuracy of the MCID estimates derived from these anchors. However, the use of two anchors and the distribution method to determine MCID provided a robust approach and enabled us to sense-check our results. Future studies would benefit from assessing the MCID at three months and at later follow-up periods to evaluate potential variability over time. Furthermore, larger cohorts and alternative anchors, especially for step count, are needed to confirm MCID.

## Conclusion

This study highlights that even small improvements in walking capacity and daily step count can be meaningful for patients recovering from LSS surgery. The identified MCIDs for the 6MWD (26–35 m) and daily step count (680–750 steps) provide practical thresholds for assessing meaningful change and can be used to inform goal setting within rehabilitation.

The variability in MCID estimates based on calculation methods reinforces the need for context-specific thresholds in clinical practice and research. While further research is needed to validate the MCID for daily step count, the data presented offer indicative benchmarks for future studies. These thresholds can also inform the design of clinical trials and the evaluation of new interventions, ensuring reported outcomes are both statistically and clinically significant.

## Electronic supplementary material

Below is the link to the electronic supplementary material.

Supplementary Material 1

## Data Availability

The datasets generated and analyzed during the current study are available from the corresponding author on reasonable request.
