# Supplementary material for: Minimal clinically important difference of the 6-Minute walk test and daily step count at 3 months following surgery for lumbar spinal stenosis
Source: Eur Spine J. 2025 Jul 15;34(12):5690–700. doi: 10.1007/s00586-025-09085-4 (PMC12714853; doi:10.1007/s00586-025-09085-4)

Minimal Clinically Important Difference of the 6-Minute Walk Test and Daily Step Count at 3 Months following Surgery for Lumbar Spinal Stenosis

Supplementary materials

Fig. 1 Histograms illustrating the distribution of the 6MWD change scores for responder and non-responders a) anchored to the ODI; b) anchored to satisfaction with walking

1a. anchored to the ODI

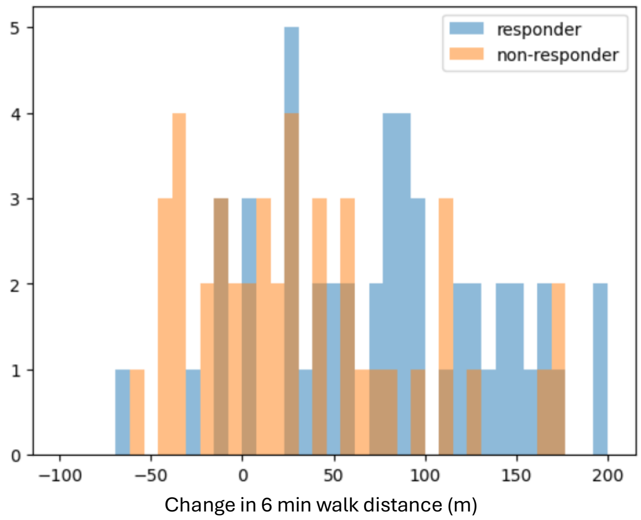

1b. anchored to walking satisfaction

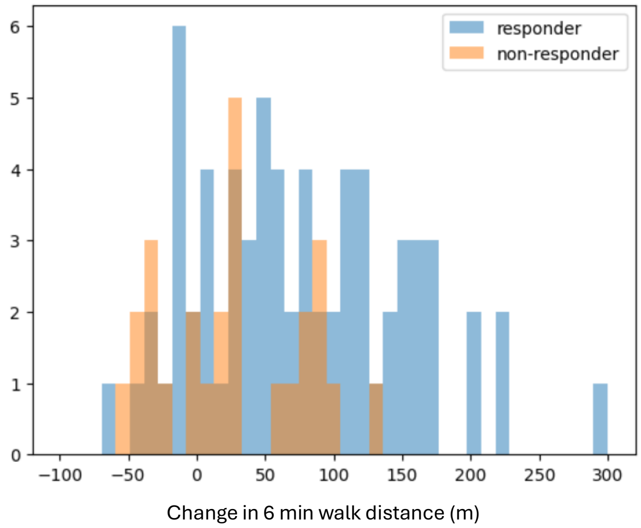

Fig. 2 Histograms illustrating the distribution of the mean daily step count change values for responder and non-responders a) anchored to the ODI; b) anchored to satisfaction with walking

2a. anchored to the ODI

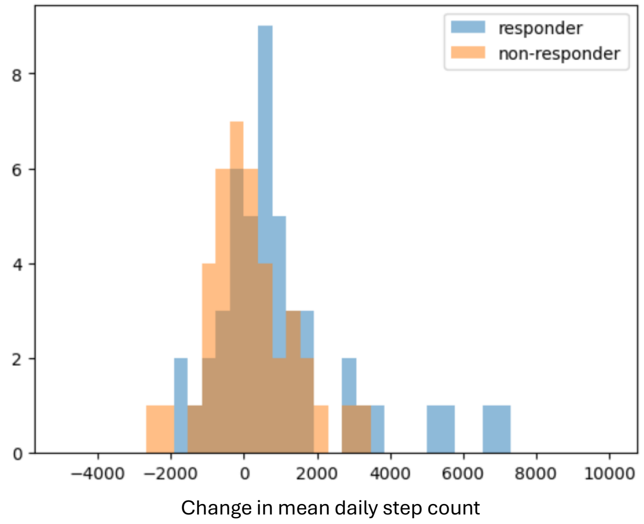

2b. anchored to walking satisfaction

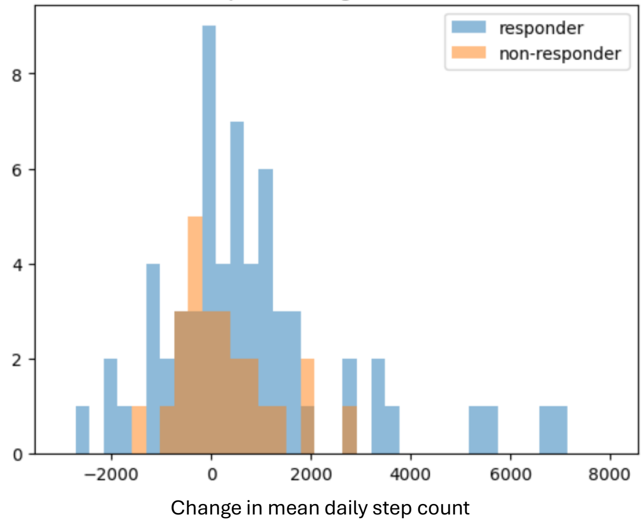

Fig. 3 illustrating the pre- and post-surgical walking measures for the whole cohort a) 6MWD, b) step count

a.

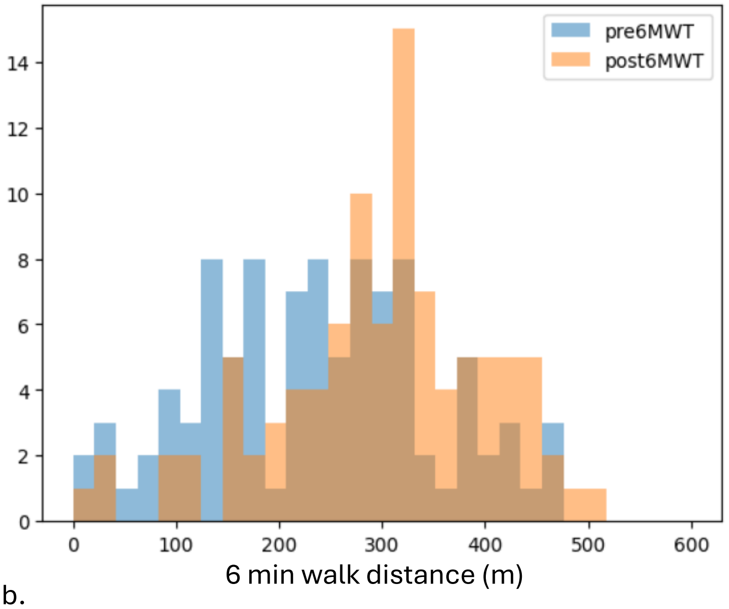

b.

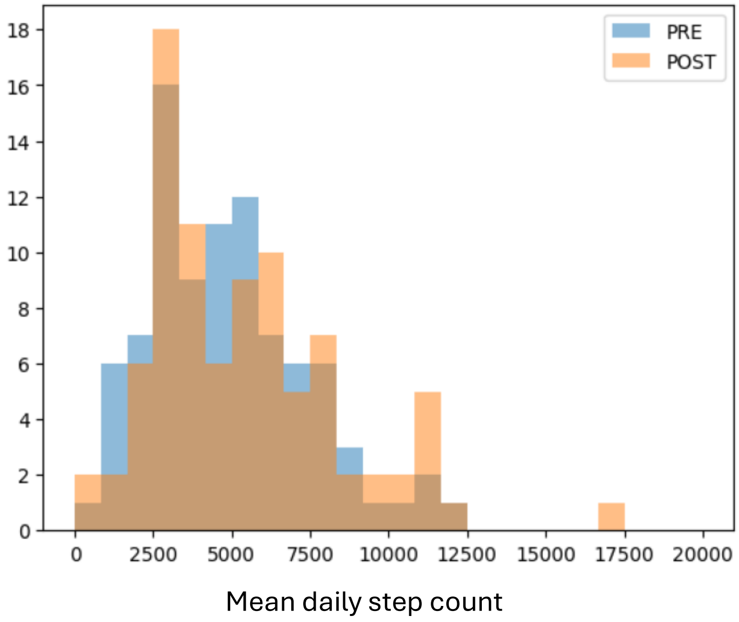

Fig. 4 scatterplots illustrating change in walking measures compared to change in ODI and post-op walking satisfaction

a. Change in 6MWD

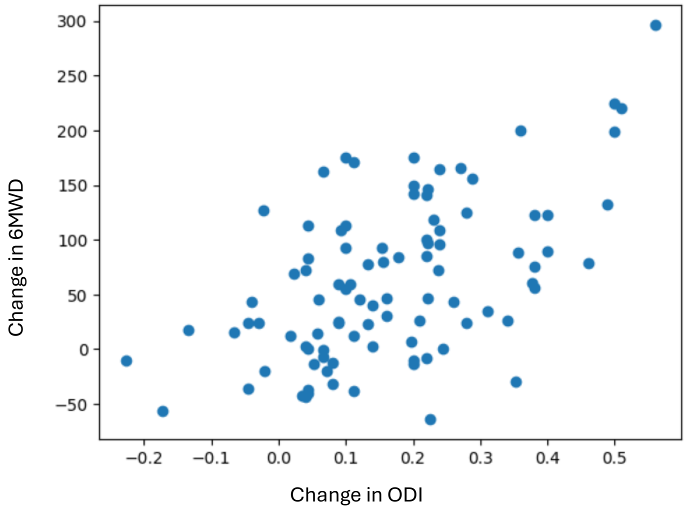

b. Change in mean daily step count

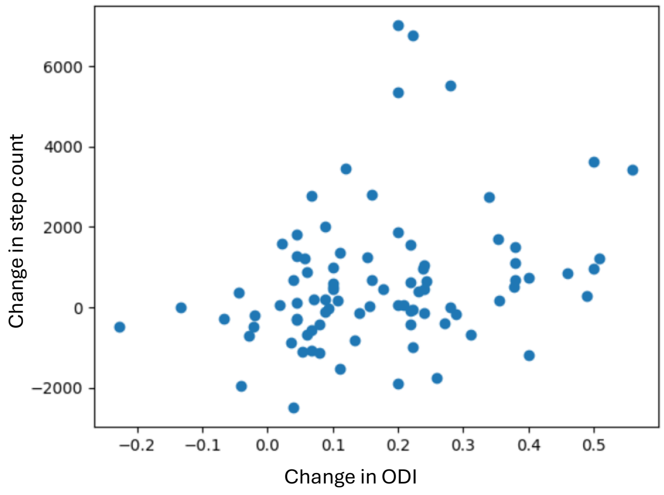

c. Change in 6MWD vs post-op walking satisfaction

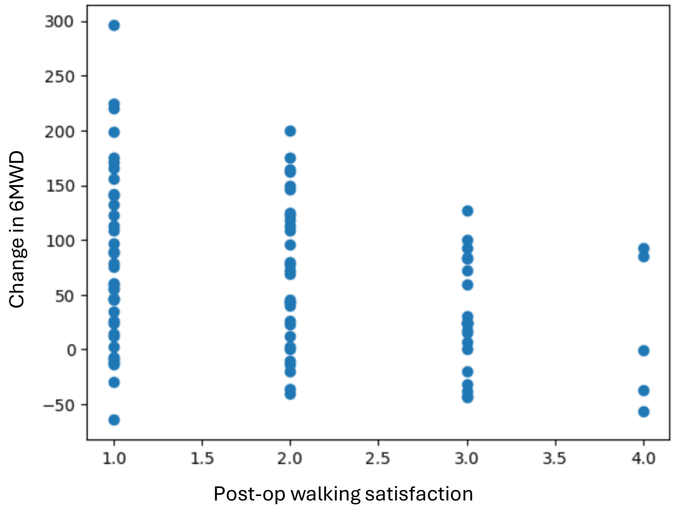

Supplement: Supplementary file 1 — Supplementary Material 1 [file 586_2025_9085_MOESM1_ESM.pdf]
